# Mathematical Methods and Applications in Medical Imaging 2014

**DOI:** 10.1155/2015/685036

**Published:** 2015-05-20

**Authors:** Liang Li, Tianye Niu, Yi Gao

**Affiliations:** ^1^Department of Engineering Physics, Tsinghua University, Beijing 100084, China; ^2^Key Laboratory of Particle & Radiation Imaging (Tsinghua University), Ministry of Education, Beijing 100084, China; ^3^Sir Run Run Shaw Hospital and Institute of Translational Medicine, Zhejiang University, Hangzhou, Zhejiang 310000, China; ^4^Departments of Biomedical Informatics, Computer Science, and Applied Mathematics and Statistics, Stony Brook University, Stony Brook, NY 11794-8322, USA

Medical imaging applies different techniques to acquire human images for clinical purposes, including diagnosis, monitoring, and treatment guidance. As a typical multidisciplinary field, medical imaging requires the improvements in both science and engineering to implement and maintain its noninvasive feature. Computational and mathematical methods are involved with imaging theories, models, reconstruction algorithms, image processing, quantitative imaging techniques, acceleration techniques, and multimodal imaging techniques. The main purpose of this issue is to bridge the gap between mathematical methods and their applications in medical imaging. This special issue covers most of the common medical imaging modalities, such as CT, PET, SPECT, MRI, ultrasound, phase contrast, and various image processing methods, such as segmentation, registration, fusion, identification, and denoising.

This special issue has 21 papers which were reviewed by at least two reviewers. Seven papers are involved with medical image reconstruction. Few-view CT scanning is a promising low-dose CT imaging mode in applications. B. Yan et al. propose an iterative few-view CT image reconstruction based on nonuniform fast Fourier transform and alternating direction total variation minimization. Different from Yan's algorithm, H. Qi et al. propose an adaptive T*p*V regularization algorithm which uses variable *p* value instead of the traditional constant* p*-norm of the image gradient magnitude. M. Brambilla et al. assess the robustness and reliability of an adaptive thresholding algorithm for the Biological Target Volume estimation incorporating PET reconstruction parameters. M. Chang et al. study the automatic exposure control strategies and propose a few-view prereconstruction guided tube current modulation method by keeping the SNR of the sinogram proximately invariable. J. Jang et al. propose a reconstruction method to quantify the distribution of blood flow velocity fields, a potentially useful index of cardiac dysfunction, inside the left ventricle from color flow ultrasound images. X-ray grating interferometry offers more information compared with traditional X-ray attenuation imaging especially for the study of weakly absorbing samples. X. Jiang et al. propose a low-dose differential phase reconstruction algorithm which adopts a differential algebraic reconstruction technique with the explicit filtering based sparse regularization rather than the common total variation. B. Wang and L. Li write a review paper on recent developments of dual-dictionary learning method in medical image analysis and reconstruction, which also discusses its role in the future studies and potential applications in medical imaging.

As a typical and important application, there are 13 papers involved with various medical image processing methods including segmentation, registration, fusion, denoising, and detection. F. Akram et al. present a region based image segmentation algorithm using active contours with signed pressure force function. It has the potential to contemporaneously trace high intensity or dense regions in an image by evolving the contour inwards. Local feature calculation is important for delineation of hippocampus, a well-known biomarker for Alzheimer disease and other neurological and psychiatric diseases. S. Tangaro et al. compare four different techniques for feature selection from a set of 315 features extracted for each voxel. The authors obtain comparable state-of-the-art performances by using only 23 features for each voxel. M. Vlachos and E. Dermatas present a finger vein segmentation method from infrared images based on a modified separable Mumford-Shah model and local entropy thresholding method. In order to improve the performance of the registration in presence of tumor shrinkage between planning CT images and posttreatment CT images, J. Wang et al. propose a registration method by combining an image modification procedure and a fast symmetric Demons algorithm. L. Zhao and K. Jia propose a diffeomorphic image registration algorithm for capturing large and complex deformation by using a two-layer deep adaptive registration framework. S. Mazaheri et al. present an ultrasound image fusion method which weights the image information within the overlapping regions by using a combination of principal component analysis and discrete wavelet transform. It is expected to increase the segment-ability of echocardiography features and decrease impact of noise and artifacts. For the purpose of quantitative analysis of the dynamic behavior about membrane-bound secretory vesicles, J. Wu et al. present a method to automatically identify the fusion events between VAMP2-pHluorin labeled GLUT4 storage vesicles and the plasma membrane in TIRF microscopy image sequences. J. Zhang et al. present a method automatically detecting the hinge point of mitral annulus in echocardiography by combining local context feature with additive support vector machines classifier. R. Xiao et al. present a seed point detection method by using adaptive ridge point extraction for coronary artery segmentation in X-ray angiogram image. L. Liu et al. present an adhesion pulmonary nodule detection method for 2D lung CT images based on the dot-filter and centerline extracting algorithm. R. Takalo et al. present an improved autoregressive model to reduce noise in SPECT images. This AR filter may be applied in both projection image and SPECT reconstruction image filtration. Nonlocal means filtering is an effective algorithm to remove the mottled noise by using large-scale similarity information in low-dose CT. However, it is very time-consuming. L. Zhang et al. present an optimized parallelization method for NLM filtering by avoiding the repeated computation with row-wise intensity calculation and the symmetry weight calculation. M. Martin-Fernandez and S. Villullas propose a MRI image denoising method by performing a shrinkage of wavelet coefficients based on the conditioned probability of noise. Instead of using an estimator of noise variance, its parameters are calculated by means of the Expectation Maximization method. B. Yu et al. present a method on estimating the binomial proportions of sensitive or stigmatizing attributes in the population of interest in successive sampling on two occasions.

We hope that this special issue may represent the state of the art and would attract wide attention of the researchers in medical imaging field.

